# Systematic synthesis of rare sugars and stereospecific conversion via photocatalysis

**DOI:** 10.1038/s41598-025-02758-6

**Published:** 2025-05-28

**Authors:** Pratiksha Babgonda Patil, Sho Usuki, Naoko Taki, Yuma Uesaka, Sanjay S. Latthe, Shanhu Liu, Kenji Yamatoya, Kazuya Nakata

**Affiliations:** 1 Graduate School of Bio-Applications and Systems Engineering, Tokyo University of Agriculture and Technology, 2-24-16 Naka-cho, Koganei, Tokyo, 184-0012 Japan; 2 Vivekanand College, C.S. No 2130 E Ward, Tarabai Park, Kolhapur, Maharashtra 416 003 India; 3 Henan Joint International Research Laboratory of Environmental Pollution Control Materials, Henan Key Laboratory of Polyoxometalate Chemistry, College of Chemistry and Chemical Engineering, Henan University, Kaifeng, 475004 People’s Republic of China; 4 Laboratory of Genomic Function Engineering, Department of Life Sciences, School of Agriculture, Meiji University, 1-1-1 Higashimita, Tama-ward, Kawasaki, 214-8571 Kanagawa Japan

**Keywords:** Photocatalysis, Photocatalysis

## Abstract

Rare sugars have gained attention as potential raw materials for pharmaceuticals and functional foods. Photocatalysis presents a promising approach for rare sugar synthesis due to its mild reaction conditions and environmental compatibility. While previous photocatalytic methods reported individual routes for specific rare sugars, systematic synthesis through a unified methodology has not been achieved, and control of optical isomers remains insufficiently explored. This study investigated a comprehensive photocatalytic approach for the systematic conversion of monosaccharides with stereochemical configuration preservation. When d-glucose in aqueous solution underwent photocatalytic treatment under UV irradiation, d-arabinose formation was confirmed through HPLC, LCMS, and ¹H NMR analyses. Similarly, d-lyxose, d-ribose, and d-xylose were produced from d-galactose, d-allose, and d-gulose, respectively. Further photocatalytic treatment of these aldopentoses yielded corresponding aldotetroses—d-erythrose from d-ribose and d-arabinose, and d-threose from d-lyxose and d-xylose. This demonstrated successful systematic conversion from aldohexoses to aldopentoses and subsequently to aldotetroses in a single reaction system. Moreover, when l-glucose and l-arabinose were used as starting materials, l-arabinose and l-erythrose were obtained, respectively, confirming stereochemical configuration preservation throughout the conversion process. This method provides a systematic approach for rare sugar synthesis while controlling stereochemical configurations.

**Supplementary Information:**

The online version contains supplementary material available at 10.1038/s41598-025-02758-6.

##  Introduction

Among the diverse monosaccharides found in nature, only seven (d-glucose, d-galactose, d-mannose, d-ribose, d-xylose, d-fructose, and l-arabinose) are naturally abundant^1^. The remaining monosaccharides are classified as “rare sugars,” which are defined as monosaccharides and their derivatives that exist in trace quantities in nature^2^. In recent years, rare sugars have garnered attention for their potential applications as materials in pharmaceuticals and foods^3–5^. For instance, allose, a representative rare sugar, has been reported to exhibit various physiological activities, including suppression of reactive oxygen species production^6^, inhibition of cancer cell proliferation^7^, and prevention of hypertension development^8^. Additionally, psicose demonstrates pharmaceutical efficacy and possesses 70% of the sweetness of sugar while being zero-calorie^9^, along with having protein antioxidant properties^10^, anti-atherosclerotic effects^11^, and anti-obesity properties^10^, making it promising for applications in the food industry.

Research on rare sugar synthesis has progressed remarkably in recent years, with biological methods garnering particular attention^12,13^. Izumoring is one of the most representative synthetic approaches^14^. This method is a comprehensive process combining aldose-ketose isomerization reactions, ketose C-3 epimerization reactions, and monosaccharide oxidation-reduction reactions. Through the skillful utilization of four enzymes—d-tagatose 3-epimerase, aldose isomerase, oxidoreductase, and aldose reductase—this method enables the production of all hexoses^14^. Furthermore, the multi-enzyme catalytic pathway utilizing dihydroxyacetone phosphate (DHAP)-dependent aldolases have opened new possibilities in rare sugar synthesis. These enzymes catalyze aldol reactions, producing rare sugars and their derivatives through the combination of DHAP with various aldehydes^14^ Although this method faces challenges regarding enzyme stability and cost, it holds great promise owing to its specificity and efficiency. Microbial synthesis methods have also been developed^15,16^. For example, a method using dihydroxyacetone phosphate-dependent aldolase from *Corynebacterium glutamicum* has been reported for the synthesis of rare sugars from glycerol^17^. Additionally, a fermentation system using *Escherichia coli* BL21 (DE3) was developed to successfully produce rare sugars, such as d-allulose and d-sorbose, from glycerol^18^.

Chemical approaches to rare sugar synthesis have also made significant advances in recent years^19,20^. One notable example is the selective epimerization reaction using Ru(II) catalyst and boron^21^. This method enables efficient conversion of 1,2-trans-diols to 1,2-cis-diols. The reaction mechanism involves controlling the equilibrium through boronic ester formation to generate the desired 1,2-cis-diol products. This single-step process is highly efficient and allows the direct use of products for further functionalization and glycosylation, thereby providing diverse approaches for constructing sugar chain structures in natural products and bioactive compounds. Treatment with subcritical aqueous ethanol has also garnered attention as a novel method for rare sugar synthesis^22^. This method enables the synthesis of rare sugars such as d-tagatose, d-xylulose, and d-ribulose using common aldoses such as d-glucose as starting materials. This approach offers the potential for a more environmentally friendly and efficient process compared to conventional methods. Additionally, rare sugars can be synthesized through isomerization and epimerization in compressed, high-temperature phosphate buffer solutions^23^. In this method, sugars, such as galactose and glucose, are processed at high temperatures to produce rare ketoses and epimers. In addition to these methods, direct C-H activation strategies have also emerged as a powerful tool in carbohydrate chemistry, proving particularly useful for the synthesis of certain rare sugars, including access to various l-hexoses^24^.

Photocatalysts are materials that promote oxidation-reduction reactions by absorbing light energy, and they have garnered attention as materials for environmental purification and organic synthesis. In typical semiconductor photocatalysts, such as TiO_2_, there is a band gap between the valence and conduction bands. When the photon energy exceeds this bandgap, electrons are excited from the valence band to the conduction band, simultaneously generating holes in the valence band. The electron-hole pairs generated in this process react with chemical species adsorbed on the surface, initiating oxidation-reduction reactions^25,26^. A notable characteristic of photocatalytic reactions is the mild reaction conditions. Compared to conventional thermal catalytic reactions, photocatalytic reactions can be conducted near room temperature, making them applicable to the transformation of heat-sensitive compounds, such as biological substances.

Several reports have demonstrated the synthesis of rare sugars using photocatalysis^27–30^. For example, in the photocatalytic conversion of glucose using gold nanoparticle-supported TiO_2_, irradiation with visible light achieved a maximum glucose conversion and gluconic acid yield of 99%^31^. In this process, the localized surface plasmon resonance effect of gold nanoparticles plays a crucial role, promoting electron transfer to TiO_2_ and enabling selective glucose oxidation through the generation of active superoxide radicals. In another example, using a Bi_2_WO_6_/CopZ composite as a photocatalyst, the selective photocatalytic oxidation of glucose to arabinose was demonstrated under visible-light irradiation and atmospheric oxygen^32^. Under the optimized conditions, the total selectivity for arabinose and formic acid reached 96.8%, with a glucose conversion of 45.3%. These studies demonstrate the potential revolutionary role of photocatalysts in rare sugar synthesis. Photocatalysts not only enable efficient conversion under mild conditions but also offer advantages over enzymes in terms of durability and reusability, while avoiding the use of environmentally harmful reagents employed in some chemical methods.

Saccharides exist as d- and l-optical isomers, with d-forms predominating in nature. The stereochemical distinction between the d- and l-forms has significant implications for physiological activities and metabolic processes^33,34^. Enzymes exhibit strict stereospecific recognition of their substrates, resulting in markedly different reactivity between the d- and l-forms^35^. Stereochemical control is crucial in pharmaceutical development because differences in drug stereoisomers can significantly influence both therapeutic efficacy and toxicity profiles^36^. For rare sugar synthesis, maintaining the d- or l-configuration of the starting materials is essential, as it directly impacts the stereochemical purity of the products and their potential biological activities.

In conventional photocatalytic synthesis methods, although individual synthetic routes have been reported for each rare sugar^37–39^, there has been no comprehensive approach capable of synthesizing various rare sugars using a single method. In other words, each rare sugar required its own “recipe,” and there is no unified method to handle them collectively. Furthermore, detailed investigations of the optical isomers (d- and l-forms) of the obtained rare sugars have rarely been conducted.

This research led to two significant discoveries in the photocatalytic synthesis of rare sugars. First, this photocatalytic method enabled the systematic synthesis of d-form aldopentoses and d-form aldotetroses. This generation of diverse sugars through a single reaction system represents the discovery of a “universal recipe” and marks a significant milestone in glycoscience. Second, preservation of the stereochemical configuration was confirmed, wherein d-form natural sugars produced d-form rare sugars, and l-form sugars produced l-form rare sugars. These discoveries bring revolutionary progress to the field of rare sugar synthesis.

##  Experimental section

###  Photocatalytic conversion of d-form sugars

A 50 mL solution of 15 mM d-glucose was prepared. To this d-glucose (Wako) solution, 25 mg of TiO_2_ (P25, Evonik) was added. The reaction was initiated by UV irradiation with an intensity of 10 mW cm^− 2^. The same procedure was carried out for d-galactose (Wako), d-allose (Funakoshi), d-gulose (Funakoshi), d-ribose (Wako), d-arabinose (Tokyo Chemical Industry), d-lyxose (Wako), and d-xylose (Nacalai Tesque). Samples were collected in 1 mL aliquots after UV irradiation. After centrifugation, the supernatant was collected and filtered using a syringe filter.

### Photocatalytic conversion of l-form sugars

A 50 mL solution of 15 mM l-glucose (Funakoshi) was prepared. To this l-glucose solution, 25 mg of TiO_2_ was added. The reaction was initiated by UV irradiation with an intensity of 10 mW cm^− 2^ intensity. For l-arabinose (Tokyo Chemical Industry), a 15 mM l-arabinose aqueous solution was prepared, and 25 mg of PtCl/TiO_2_ (MPT-623, Ishihara Sangyo Kaisha, Ltd.) was used as the photocatalyst. Samples were collected in 1 mL aliquots after UV irradiation. After centrifugation, the supernatant was collected and filtered using a syringe filter.

### HPLC and LCMS analysis

Prior to measurement, the product was derivatized with p-aminobenzoic acid ethyl ester (ABEE, Tokyo Chemical Industry) by the following process: ABEE solution was prepared by mixing ABEE (332 mg), sodium cyanoborohydride (31.7 mg), acetic acid (386 µL), and methanol (3.60 mL). Subsequently, 10.0 µL of a solution containing the products was mixed with 40.0 µL of the ABEE solution and vortexed for 15 s. The resulting solution was centrifuged for 2 min, then heated to 80 °C. After 1 h, the mixture was cooled to room temperature and centrifuged for 2 min. Water (200 µL) and chloroform (200 µL) were then added, the mixture was vortexed for 1 min, and subsequently centrifuged for an additional 2 min. Separate water and chloroform layers were formed, 150 µL of the upper aqueous layer was collected, and 300 µL water was added to the aqueous solution. Finally, the solution was vortexed for 1 min and centrifuged for 2 min.

The analysis of ABEE-derivatized samples were carried out using a high- performance liquid chromatography (HPLC) instrument (Shimadzu) equipped with a UV-VIS detector (305 nm). A CAPCELL PAK C18 (150 mm×4.6 mm I.D., SHISEIDO) was used for the analysis, and the temperature was maintained at 40 °C. Ammonium acetate solution was used as the eluent (20.0 mmol L^− 1^)/acetonitrile (87/13% v/v), and the flow rate was 1.0 mL min^− 1^.

Liquid chromatography mass-spectrometry (LCMS) (LCMS8050, Shimadzu) was used for the analysis of ABEE-derivatized samples. A CAPCELL PAK C18 (150 mm × 2.0 mm I.D., SHISEIDO) was used for the analysis, and the temperature was maintained at 40 °C. Ammonium acetate solution was used as the eluent (10.0 mmol L^− 1^)/acetonitrile (87/13% v/v) and the flow rates were 0.5 mL min^− 1^ for d-sugar conversion and 0.2 mL min^− 1^ for l-sugar conversion.

### ^1^H NMR measurement

After the photocatalyst powder was removed from the reaction sample using a syringe filter, the solution was concentrated to 2 mL using a rotary evaporator. The ABEE labeling reagent (3 mL) was added to 1 mL of the concentrated solution. After mixing using a vortex mixer, the mixture was heated at 80 °C for 1 h in an aluminum block thermostat bath. After cooling to room temperature, liquid-liquid extraction with chloroform (2 mL) was performed 3–5 times to remove unreacted ABEE reagent. The aqueous layer was subjected to preparative HPLC under the following conditions: column, CAPCELL PAK C18 (250 mm × 20 mm I.D., SHISEIDO); mobile phase, 20 mM ammonium acetate aqueous solution: acetonitrile = 87:13 (v/v); flow rate, 18 mL min^–1^; detection, UV 305 nm; injection volume, 1000 µL. The collected fractions were concentrated to 2 mL using a rotary evaporator and dried under nitrogen flow. When complete drying was difficult, a small amount of toluene was added for azeotropic distillation, followed by drying again under nitrogen flow. The dried material was dissolved in D_2_O containing DSS (internal standard) to prepare the sample for the ^1^H NMR measurements ^1^H NMR spectra were measured using a 500 MHz NMR spectrometer (RESONANCE / ECP-500, JEOL).

### l-tryptophanamide labeling

l-tryptophanamide labeling reagent was prepared as follows: 4 mL of 100 mmol L^–1^ sodium tetraborate (Wako) was mixed with 2 mL of methanol. l-tryptophanamide (Tokyo Chemical Industry) was then added to this solution to achieve a final concentration of 100 mmol L^–1^. The labeling procedure was performed by adding 300 µL of l-tryptophanamide labeling reagent and 100 µL of 2 mol L^–1^ dimethylamine borane solution to 100 µL of the sample. The solution was then incubated at 40 °C for 4 h. After incubation, the solution was combined with 500 µL of 1 mol L^–1^ sodium chloride (Wako Pure Chemical Industries, 99.5%) solution and subsequently diluted four-fold with carrier solution for HPLC analysis.

HPLC analysis was performed using a reversed-phase column (CAPCELL PAK C18 AQ, column 150 × 4.6 mm I.D., OSAKASODA) with detection at 220 nm using a UV detector (SPD-20 A, Shimadzu). The column temperature was maintained at 40 °C with a flow rate of 1.0 mL min^–1^. The carrier solution was prepared by mixing 10 mmol L^–1^ ammonium acetate buffer (pH 7.6) containing 1.5 mmol L^–1^ butylboronic acid (Tokyo Chemical Industry) and acetonitrile in a volume ratio of 95:5.

## Results and discussion

**Fig. 1:**
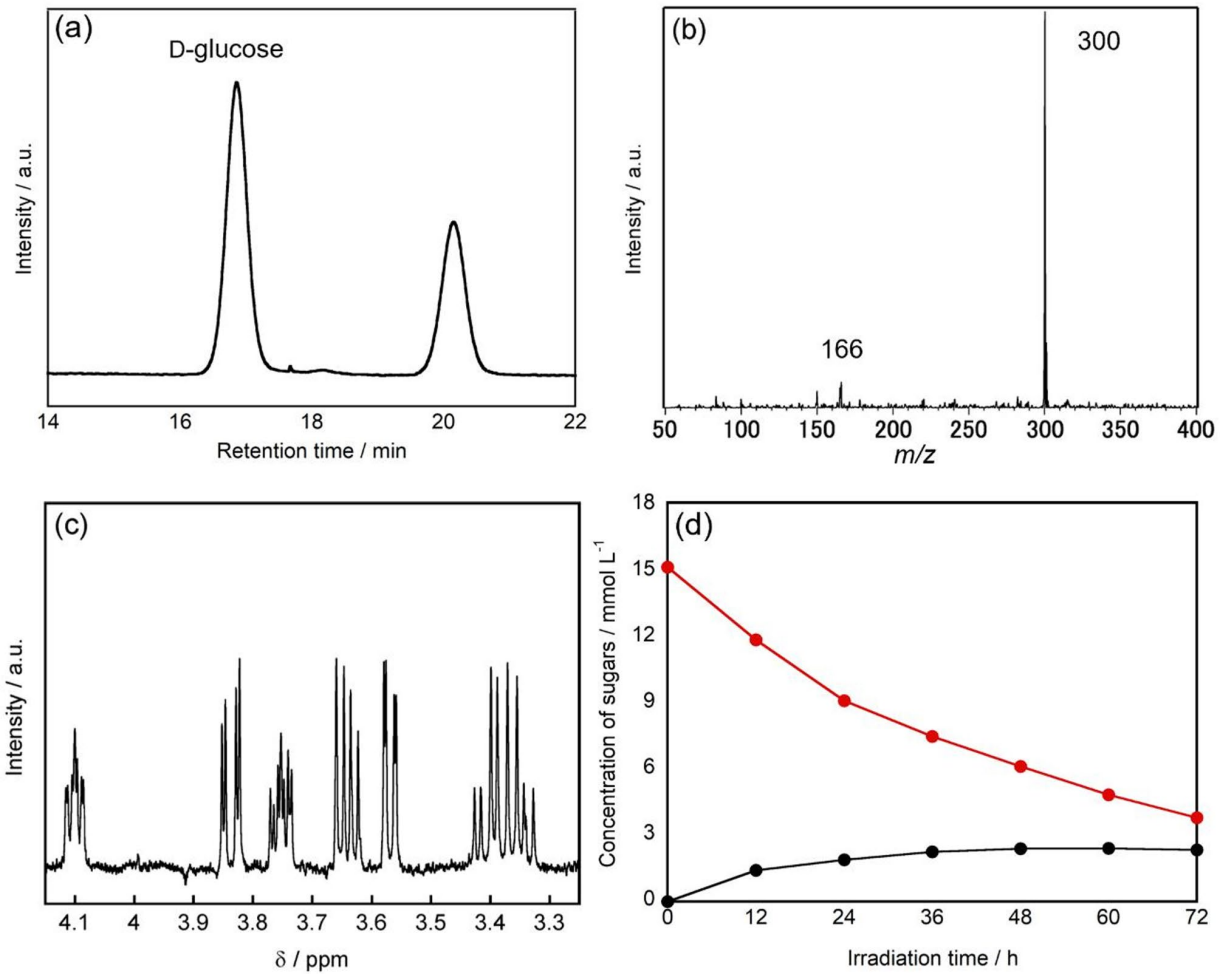
(**a**) HPLC chromatograms of the sample obtained after TiO_2_ treatment of d-glucose after 72 h of UV irradiation. (**b**) Mass spectrum of the isolated sample found in the R.T. = 20.6 min in HPLC analysis. (**c**) ^1^H NMR spectrum of the obtained sample. (**d**) Changes in d-glucose (red) and d-arabinose (black) concentrations as a function of irradiation time.

D-glucose dissolved in water was treated with the photocatalyst under UV irradiation at room temperature in air. Representative chromatograms highlighting the significant peaks are presented in the main text, while complete chromatographic profiles are provided in Figure S1. Figure 1a shows the HPLC chromatogram of the sample after 72 h of treatment. The peaks at retention times (R.T.) of 17.0 min and 20.6 min corresponded to those of d-glucose and d-arabinose standard materials, respectively.

To further identify the products, molecular weight measurements were performed using LCMS. In this study, the molecular weights of the products were measured in the ABEE-labeled state. Therefore, the observed molecular weights were calculated as follows: (observed molecular weight) = (molecular weight of the products) + (molecular weight of ABEE, 165)—(atomic weight of oxygen lost during bonding, 16) + (atomic weight of H added during ionization, 1)^40^. For the products obtained using the photocatalyst, the molecular weight of the peak with a retention time of 20.6 min was measured using LCMS, resulting in *m/z* = 300 (Fig. 1b). Considering the addition of ABEE and ionization, the molecular weight of this product was 300–165 + 16–1 = 150. This corresponded to the molecular weight of d-arabinose.

To determine the structure of compounds produced by the photocatalytic reaction of d-glucose, ^1^H NMR analysis was performed (Fig. 1c). The ^1^H NMR spectrum of the compound with a retention time of 20.6 min, isolated and purified by preparative HPLC, matched completely with that of the d-arabinose standard material. These results demonstrate that d-arabinose was produced during the photocatalytic reaction of d-glucose.

Changes in the concentrations of compounds in the reaction solution were monitored during the photocatalytic reaction of d-glucose. Figure 1d shows the changes in d-glucose and d-arabinose concentrations as a function of UV irradiation time. The initial concentration of d-glucose was 15.2 mmol L^–1^, which gradually decreased with UV irradiation, reaching 3.8 mmol L^–1^ after 72 h. This decrease indicates the decomposition of d-glucose through a photocatalytic reaction under UV irradiation. Meanwhile, the concentration of the product d-arabinose gradually increased, reaching 2.4 mmol L^–1^ after 72 h. These results demonstrated that the conversion from d-glucose to d-arabinose proceeded progressively in this photocatalytic reaction system.

**Fig. 2:**
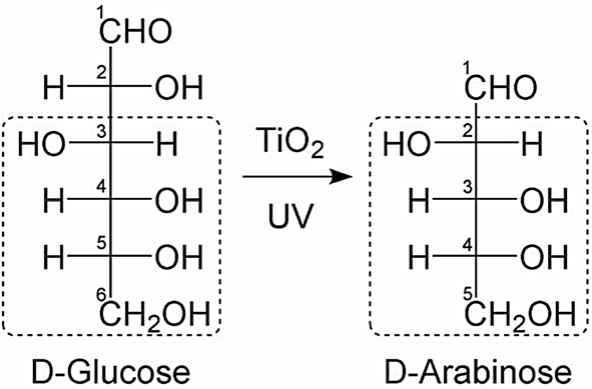
Schematic illustration of stereochemical consideration in photocatalytic transformation from d-glucose to d-arabinose.

Based on the obtained experimental results, the conversion reaction from d-glucose to d-arabinose by photocatalysis was examined. d-glucose is an aldohexose, whereas d-arabinose is an aldopentose. As shown in Fig. 2, a comparison of their molecular structures confirmed that the stereochemical configuration from the C3 to C6 positions of d-glucose was retained as the C2 to C5 structure in the product d-arabinose. This structural feature suggests that a reaction involving carbon-number reduction proceeded, involving cleavage of the C1-C2 bond in glucose, formation of an aldehyde group at the C2 position, and elimination of the C1 position to generate arabinose. To verify the generality of this conversion process, further photocatalytic reactions were conducted using various aldohexoses similar to d-glucose as substrates.

**Fig. 3:**
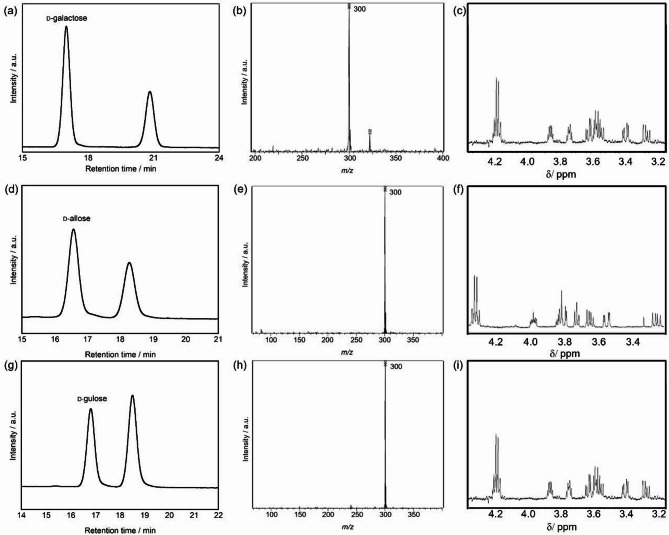
Analysis of d-galactose photocatalytic treatment: (**a**) HPLC chromatogram after 72 h of UV irradiation, (**b**) Mass spectrum of the isolated sample at R.T. = 20.8 min, and (**c**) ^1^H NMR spectrum of the obtained sample. Analysis of d-allose photocatalytic treatment: (**d**) HPLC chromatogram after 72 h of UV irradiation, (**e**) Mass spectrum of the isolated sample at R.T. = 18.3 min, and (**f**) ^1^H NMR spectrum of the obtained sample. Analysis of d-gulose photocatalytic treatment: (**g**) HPLC chromatogram after 72 h of UV irradiation, (**h**) Mass spectrum of the isolated sample at R.T. = 18.5 min, and (**i**) ^1^H NMR spectrum of the obtained sample. All samples were obtained after TiO_2_ treatment under UV irradiation for 72 h.

**Fig. 4:**
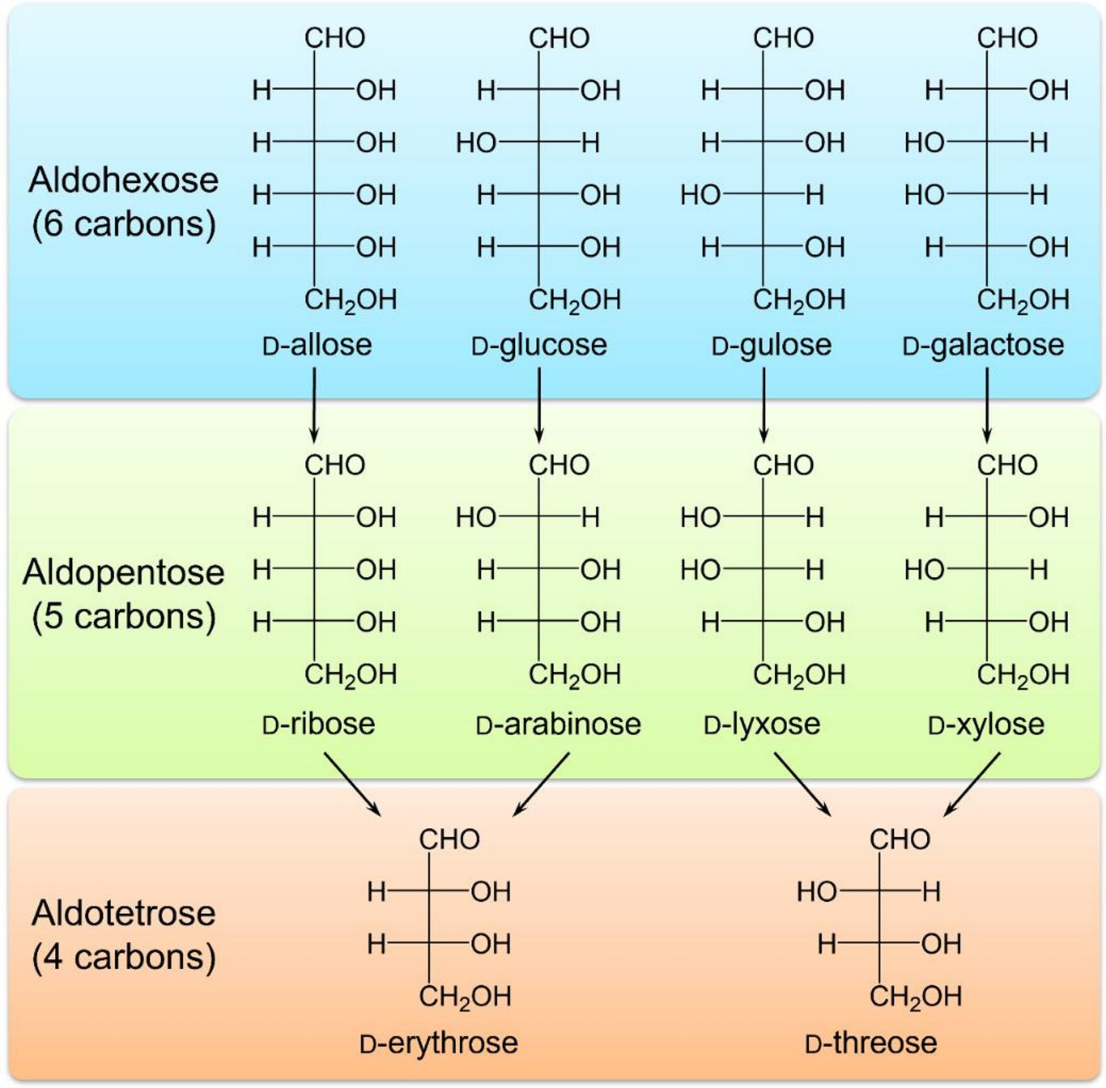
Schematic illustration of photocatalytic production of monosaccharides.

The products from photocatalytic decomposition reactions of various aldohexoses besides d-glucose were identified. Photocatalytic reactions were conducted under the same conditions for d-galactose, d-allose, and d-gulose. HPLC analysis revealed new peaks in each reaction solution with different retention times from the starting materials, which corresponded to the retention times of standard materials of aldopentoses: d-lyxose, d-ribose, and d-xylose, respectively (Fig. 3). LCMS analysis of these new peaks revealed ion peaks at *m/z* = 300 ([M + H]^+^) only or *m/z* = 300 ([M + H]^+^) and 322 ([M + Na]^+^), suggesting the formation of aldopentoses (Fig. 3). To determine the detailed structures of the products, each peak was isolated and purified using preparative HPLC and subjected to^1^H NMR analysis. The ^1^H NMR spectra of each product matched those of the corresponding standard materials, confirming the formation of d-lyxose from d-galactose, d-ribose from d-allose, and d-xylose from d-gulose (Fig. 3). The results demonstrated that the photocatalytic decomposition of aldohexoses consistently yielded corresponding aldopentoses (Fig. 4). Notably, as in the case of d-glucose, the stereochemical configuration from C3 to C6 in each aldohexose was retained as C2 to C5 in the corresponding aldopentose. This result strongly suggests that the reaction proceeds through a carbon-reduction mechanism involving selective cleavage of the C1-C2 bond.

Based on the regular transformation reactions from photocatalytic decomposition of aldohexose to aldopentose, we further investigated the generality of this reaction mechanism by examining the photocatalytic decomposition of aldopentoses. When d-ribose, d-arabinose, d-lyxose, and d-xylose were used as substrates under the same reaction conditions, HPLC analysis revealed new peaks in each reaction solution that were distinct from the starting materials. These peaks corresponded to the retention times of d-erythrose for reactions with d-ribose and d-arabinose, and d-threose for reactions with d-lyxose and d-xylose (Figure S2). LCMS analysis of these new peaks revealed ion peaks at *m/z* = 270 ([M + H]^+^), suggesting the formation of compounds with aldotetrose structures (Figure S2). The structures of the products were determined by isolating and purifying each peak using preparative HPLC, followed by ^1^H NMR analysis. The results confirmed the formation of d-erythrose from d-ribose and d-arabinose, and d-threose from d-lyxose and d-xylose (Figure S2). These results demonstrate that the photocatalytic decomposition of aldopentoses follows the same systematic carbon-reduction reaction observed for aldohexoses, yielding aldotetroses with retained stereochemical configurations (Fig. 4). This finding indicated that the carbon reduction mechanism involving selective C1-C2 bond cleavage can be applied to monosaccharides with different carbon numbers.

As mentioned above, the formation of d-arabinose from d-glucose through a photocatalytic reaction was confirmed by HPLC, LCMS, and ^1^H NMR analyses. Monosaccharides exist as optical isomers in the d- and l-forms, and this stereochemical configuration is critical for physiological activity and metabolic processes^41–43^. Therefore, it was necessary to rigorously verify the stereochemical configuration before and after the photocatalytic reaction. However, the d- and l-forms of monosaccharides have similar properties, making their separation difficult using conventional HPLC analysis. Thus, analysis using l-tryptophanamide labeling was conducted. This method enables separation on a reversed-phase column by converting d- and l-forms into diastereomers through labeling the reducing end of monosaccharides with l-tryptophanamide^44^.

**Fig. 5:**
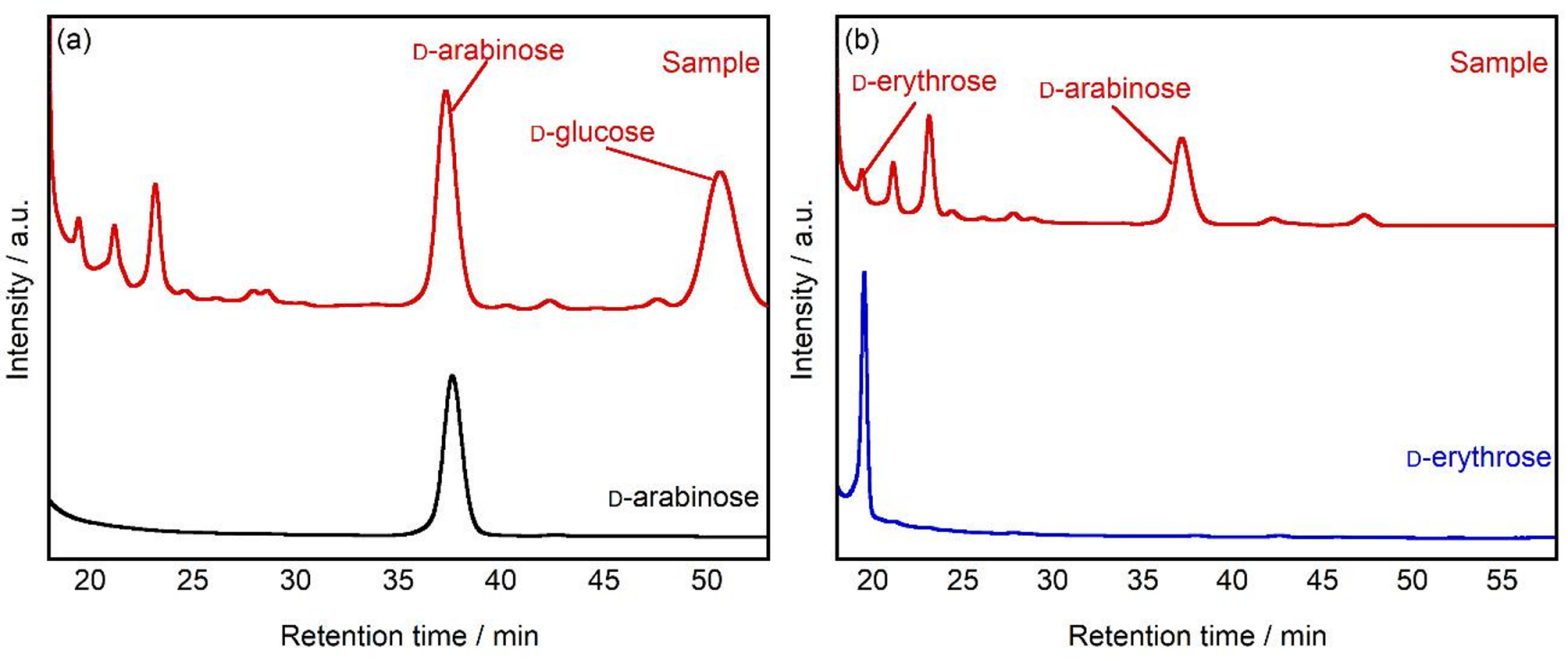
HPLC chromatograms for obtained sample after TiO_2_ treatment of (**a**) d-glucose and (**b**) d-arabinose after 72 h of UV irradiation. The products were functionalized with l-tryptophanamide. d-glucose: 15 mmol L^− 1^, TiO_2_: 25 mg, UV light: 10 mW cm^− 2^, temperature: 25 °C, column: CAPCELL PAK C18, detector: UV-VIS.

When the product obtained from the 72 h photocatalytic treatment of d-glucose was labeled with l-tryptophanamide and analyzed by HPLC, a peak was observed at a retention time 37.3 min (Fig. 5a). This retention time perfectly matched that of the d-arabinose standard material. This result definitively proves that the arabinose produced from d-glucose through the photocatalytic reaction maintains the d-form stereochemical configuration.

Furthermore, a similar analysis was performed on the erythrose produced by the photocatalytic treatment of d-arabinose. A peak was observed at a retention time of 19.4 min, which matched that of the d-erythrose standard material (Fig. 5b). This demonstrated that the stereochemical configuration was also maintained during the conversion of d-arabinose to erythrose. This preservation of the stereochemical configuration represents a significant finding, indicating that stereochemical purity control of products is possible in rare monosaccharide synthesis through photocatalysis.

**Fig. 6:**
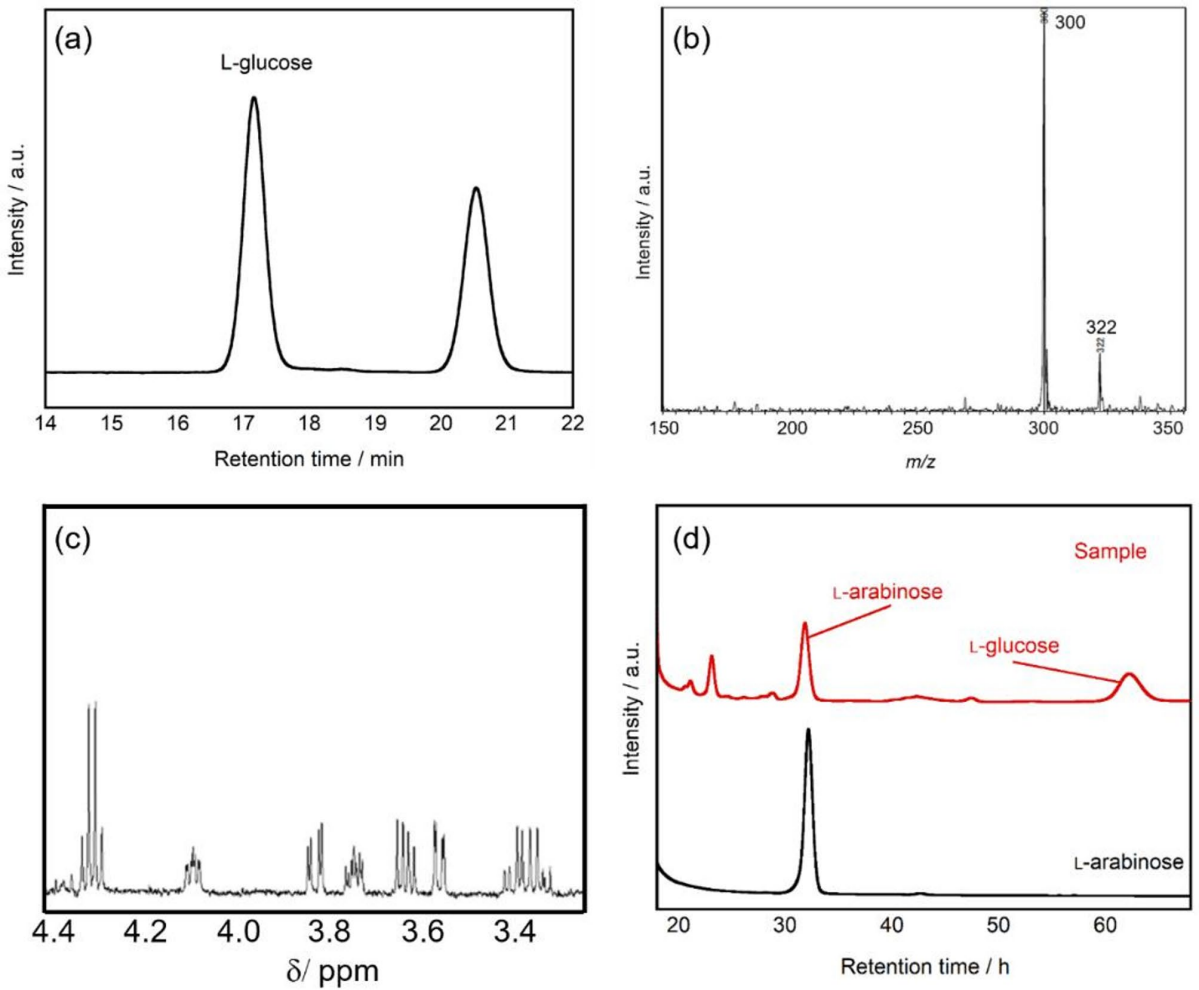
(**a**) HPLC chromatograms for obtained sample after TiO_2_ treatment of l-glucose after 72 h of UV irradiation. Products are functionalized with ABEE. l-glucose: 15 mmol L^− 1^, TiO_2_: 25 mg, UV light: 10 mW cm^− 2^, temperature: 25 °C, column: CAPCELL PAK C18, detector: UV-VIS. (**b**) Mass spectrum of the isolated sample found in R.T. = 20.5 min in HPLC analysis. (**c**) ^1^H NMR spectrum of the obtained sample. (**d**) HPLC chromatograms of the sample obtained after TiO_2_ treatment of l-glucose after 72 h of UV irradiation. The products were functionalized with l-tryptophanamide. l-glucose: 15 mmol L^− 1^, TiO_2_: 25 mg, UV light: 10 mW cm^− 2^, temperature: 25 °C, column: CAPCELL PAK C18, detector: UV-VIS.

Through photocatalytic reaction, the conversion from d-glucose to d-arabinose and further from d-arabinose to d-erythrose was achieved with retention of stereochemical configuration. While this finding demonstrates the important stereoselectivity in photocatalytic conversion, it was necessary to verify whether this stereoselectivity was also applicable to l-form monosaccharides. When an l-glucose aqueous solution was treated with the photocatalyst under UV irradiation, the HPLC chromatogram after the reaction showed a new peak at a retention time of 20.5 min in addition to the starting material (Fig. 6a). To determine the structure of the product, LCMS analysis was conducted. When the product was analyzed after ABEE labeling, ion peaks at *m/z* = 300 [M + H]^+^ and *m/z* = 322 [M + Na]^+^ were detected at a retention time of 20.5 min (Fig. 6b). This indicates a molecular mass corresponding to that of aldopentose. Furthermore, to determine the detailed structure of this compound, it was isolated and purified using preparative HPLC followed by ^1^H NMR analysis. The obtained spectrum perfectly matched that of the l-arabinose standard material (Fig. 6c).

To further examine the stereochemical configuration of the arabinose produced by the photocatalytic treatment of l-glucose, the sample treated with the photocatalyst for 72 h under light irradiation was analyzed after l-tryptophanamide labeling. The resulting chromatogram is shown in Fig. 6d. A peak was confirmed at R.T. = 31.9 min, which matched the retention time of the l-arabinose standard material. These results confirmed that the arabinose produced from l-glucose through photocatalytic reaction maintained the l-form stereochemical configuration.

Subsequently, photocatalytic conversion of l-arabinose was investigated. For the conversion of l-arabinose to l-erythrose, PtCl/TiO_2_ was employed instead of TiO_2_. Preliminary experiments indicated that PtCl/TiO_2_ provided better conversion efficiency for the conversion. The catalyst loading was adjusted according to the substrate concentration. When the l-arabinose aqueous solution was treated with the photocatalyst under UV irradiation, the HPLC chromatogram after the reaction showed a new peak, in addition to the starting material (Figure S3(a)). LCMS analysis of this peak revealed an ion peak at *m/z* = 270 [M + H]^+^, indicating a molecular mass corresponding to an aldotetrose (Figure S3(b)). Furthermore, after isolation and purification by preparative HPLC, ^1^H NMR analysis confirmed that this product perfectly matched the l-erythrose standard material (Figure S3(c)).

To further examine the stereochemical configuration of the erythrose produced by the photocatalytic treatment of l-arabinose, the sample treated with the photocatalyst for 72 h under light irradiation was analyzed after l-tryptophanamide labeling. The resulting chromatogram is shown in Figure S3(d). A peak was confirmed at R.T. = 21.1 min. When the l-erythrose standard tetrose material was measured under the same conditions, its retention time matched with the peak at R.T. = 21.1 min. These results confirm that the erythrose produced from l-arabinose through the photocatalytic reaction maintained the l-form stereochemical configuration.

**Fig. 7:**
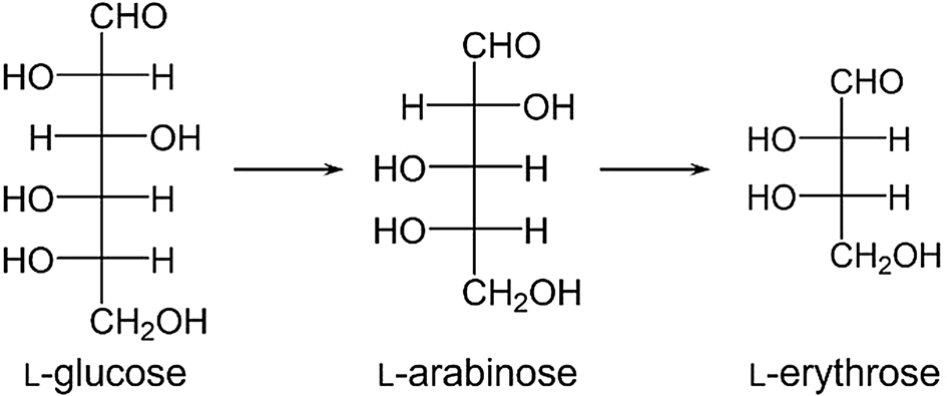
Schematic illustration of stereochemical configuration during the photocatalytic transformation pathway of l-glucose to l-arabinose and subsequent conversion to l-erythrose.

Based on these results, the conversion pathway of photocatalytic reactions with l-form monosaccharides is shown in Fig. 7. l-glucose was converted to l-arabinose through photocatalytic reaction, and l-arabinose was further converted to l-erythrose through photocatalytic reaction. This reaction pathway is completely symmetrical to the previously confirmed conversion pathway of d-form monosaccharides (d-glucose → d-arabinose → d-erythrose), clearly demonstrating retention of the stereochemical configuration in photocatalytic reactions. While the retention of configuration at remote stereocenters might be expected in some C-C cleavage reactions, experimental verification is crucial under photocatalytic conditions, which can involve reactive intermediates (e.g., radicals, reactive oxygen species) potentially leading to undesired epimerization or racemization. Confirming the stereochemical integrity using a method like l-tryptophanamide derivatization, for both d- and l-series, therefore provides essential validation for the stereospecificity claimed for this synthetic route and enhances its reliability for producing enantiopure rare sugars, which is often critical for their biological activity and applications. These findings suggest that the stereoselective synthesis of products corresponding to the stereochemical configuration of the starting materials is possible in rare monosaccharide synthesis through photocatalysis. While this study successfully demonstrates the systematic conversion pathway and its stereospecificity, we acknowledge certain limitations in the current methodology for practical application. The observed yields, such as ~ 16% for d-arabinose from d-glucose after 72 h, are modest and reflect the proof-of-concept nature of this work. The relatively long reaction times also present a challenge for practical applications. Furthermore, mass balance calculations, summarized in Table S1, indicate that the identified products account for approximately 50% of the converted substrate, suggesting that a significant portion may be converted to other byproducts, potentially including carbon dioxide. It is likely that both incomplete substrate conversion and potential side reactions, including degradation of products, contribute to the current yield limitations. Future research should focus on optimizing the reaction conditions (e.g., light source, temperature, pH, oxygen concentration), exploring alternative or modified photocatalysts (e.g., doping, morphology control, different semiconductors), investigating the use of co-catalysts, and potentially employing simulated moving bed chromatography to improve yields, enhance selectivity, and shorten reaction times. Furthermore, detailed analysis of minor byproducts and mass balance studies will be crucial for a complete understanding of the reaction network and for assessing the feasibility of scaling up this process for preparative purposes.

## Conclusions

While photocatalytic synthesis of rare monosaccharides has attracted attention due to its mild reaction conditions and environmental compatibility, conventional methods have faced significant limitations. Although individual synthetic routes have been reported for each rare monosaccharide, a comprehensive approach through a single methodology had not been achieved. Furthermore, detailed investigations regarding optical isomers (d- and l-forms) of the obtained rare monosaccharides had rarely been conducted.

In this study, two findings were made in rare monosaccharide synthesis using photocatalysis. First, the developed photocatalytic method enabled the systematic synthesis of aldopentoses and aldotetroses. The production of diverse monosaccharides through a single reaction system represents a significant milestone in glycoscience. Second, stereospecific conversion was confirmed, wherein d-form natural monosaccharides produced d-form rare monosaccharides, and l-form monosaccharides produced l-form rare monosaccharides. The term ‘stereospecific conversion’ is used here to emphasize that the reaction proceeds with complete retention of the configuration of the existing stereocenters (C3-C6 of aldohexose corresponding to C2-C5 of aldopentose), without inversion or racemization at these centers, thus producing a specific stereoisomer corresponding to the starting material’s configuration. This discovery is crucial for the control of the stereochemical purity of rare monosaccharides.

The findings of this research open new horizons in the synthesis and application of rare monosaccharides, with anticipated ripple effects extending beyond glycoscience to diverse fields including life sciences, pharmaceutical sciences, and materials science.

## Electronic supplementary material

Below is the link to the electronic supplementary material.

Supplementary Material 1

## Data Availability

The data that support the findings of this study are available from the corresponding author upon reasonable request.
