# Supplementary material for: Systematic synthesis of rare sugars and stereospecific conversion via photocatalysis
Source: Sci Rep. 2025 May 28;15:18703. doi: 10.1038/s41598-025-02758-6 (PMC12119889; doi:10.1038/s41598-025-02758-6)
Supplement: Supplementary file 1 — Supplementary Material 1 [file 41598_2025_2758_MOESM1_ESM.docx]

**Supporting information**

**Systematic Synthesis of Rare Sugars and Stereospecific Conversion via Photocatalysis**

Pratiksha Babgonda Patil^1^, Sho Usuki^1,*^, Naoko Taki^1^, Yuma Uesaka^1^, Sanjay S. Latthe^2^, Shanhu Liu^3^, Kenji Yamatoya^4,*^, Kazuya Nakata^1,*^

^1^Graduate School of Bio-Applications and Systems Engineering, Tokyo University of Agriculture and Technology, 2-24-16 Naka-cho, Koganei, Tokyo 184-0012, Japan

^2^Vivekanand College, C.S. No 2130 E ward, Tarabai Park, 416 003 Kolhapur, Maharashtra, India

^3^Henan Joint International Research Laboratory of Environmental Pollution Control Materials, Henan Key Laboratory of Polyoxometalate Chemistry, College of Chemistry and Chemical Engineering, Henan University, Kaifeng, 475004, PR China

^4^Laboratory of Genomic Function Engineering, Department of Life Sciences, School of Agriculture, Meiji University, 1-1-1 Higashimita, Tama-ward, Kawasaki 214-8571, Kanagawa, Japan

^*^Corresponding Authors

Sho Usuki, E-mail: fv7248@go.tuat.ac.jp

Kenji Yamatoya, E-mail: yamatoya_k@meiji.ac.jp

Kazuya Nakata, E-mail: nakata@go.tuat.ac.jp


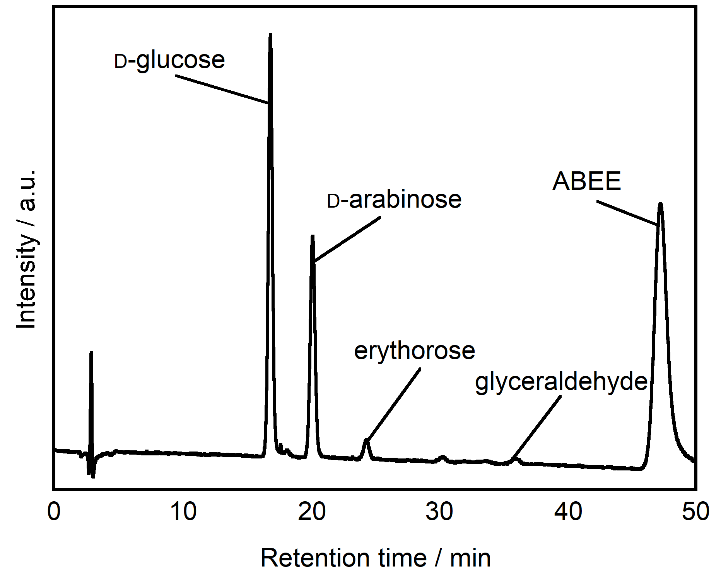


**Figure S1** HPLC chromatograms of the sample obtained after TiO_2_ treatment of d-glucose after 72 h of UV irradiation.


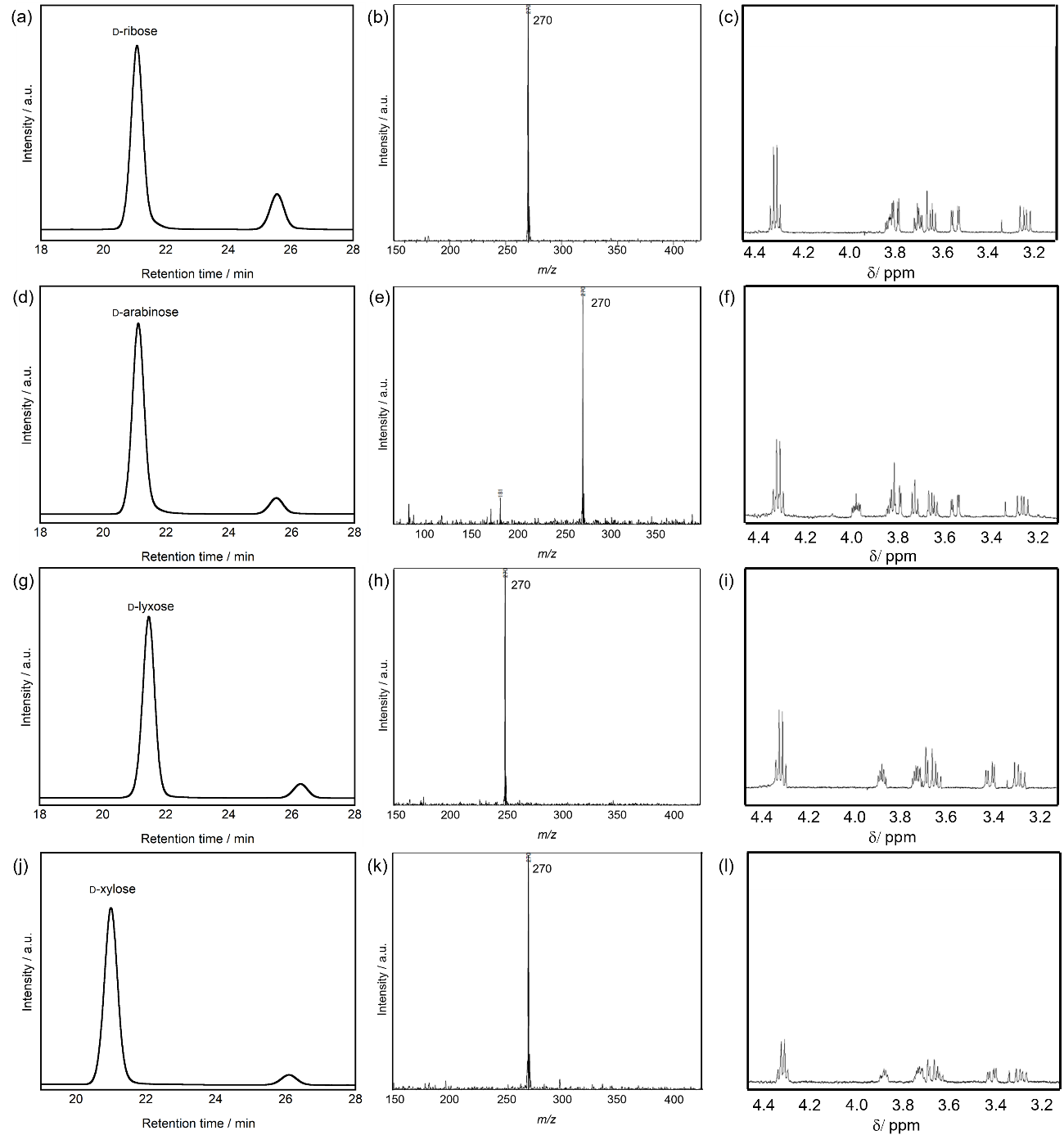


**Figure S2.** Analysis of d-ribose photocatalytic treatment: (a) HPLC chromatogram after 72 h of UV irradiation, (b) Mass spectrum of the isolated sample at R.T. = 25.6 min, and (c) ¹H NMR spectrum of the obtained sample. Analysis of d-arabinose photocatalytic treatment: (d) HPLC chromatogram after 72 h of UV irradiation, (e) Mass spectrum of the isolated sample at R.T. = 25.5 min, and (f) ¹H NMR spectrum of the obtained sample. Analysis of d-lyxose photocatalytic treatment: (g) HPLC chromatogram after 72 h of UV irradiation, (h) Mass spectrum of the isolated sample at R.T. = 26.3 min, and (i) ¹H NMR spectrum of the obtained sample. Analysis of d-xylose photocatalytic treatment: (j) HPLC chromatogram after 72 h of UV irradiation, (k) Mass spectrum of the isolated sample at R.T. = 26.1 min, and (l) ¹H NMR spectrum of the obtained sample. All samples were obtained after TiO₂ treatment under UV irradiation for 72 h.


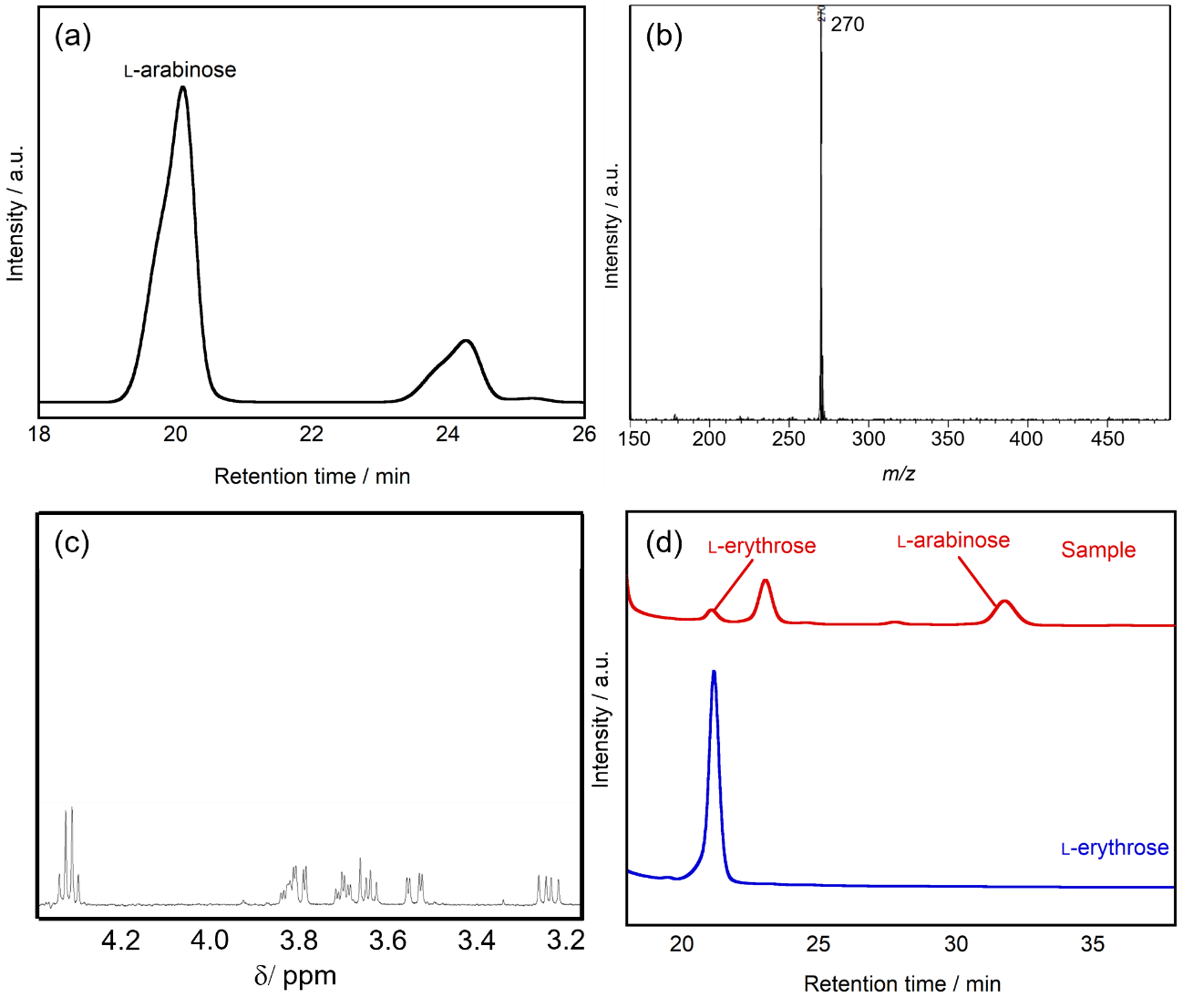


**Figure S3.** (a) HPLC chromatograms of the sample obtained after PtCl/TiO_2_ treatment of l-arabinose after 72 h of UV irradiation. Products are functionalized with ABEE. l-arabinose: 15 mmol L^-1^, PtCl/TiO_2_: 25 mg, UV light: 10 mW cm^-2^, temperature: 25ºC, column: CAPCELL PAK C18, detector: UV-VIS. (b) Mass spectrum of the isolated sample found in R.T. = 20.5 min in HPLC analysis. (c) ^1^H NMR spectrum of the obtained sample. (d) HPLC chromatograms of the sample obtained after PtCl/TiO_2_ treatment of l-arabinose after 72 h of UV irradiation. The products were functionalized with l-tryptophanamide. l-arabinose: 15 mmol L^-1^, PtCl/TiO_2_: 25 mg, UV light: 10 mW cm^-2^, temperature: 25ºC, column: CAPCELL PAK C18, detector: UV-VIS.

**Table S1.** Conversion yields of each products

| Products | Conversion yield (%) |
| --- | --- |
| Arabinose | 18.7 |
| Erythrose | 2.7 |
| Glyceraldehyde | 0.7 |
| Gluconic acid | 9.5 |
| Acetic acid | 6.0 |
| Formic acid | 11.1 |
| Total | 48.7 |
